# Supplementary material for: A Simple and Scalable Assay for Multiplexed Flow Cytometric Profiling of Surface Markers on Small Extracellular Vesicles
Source: Cells. 2025 Jun 28;14(13):989. doi: 10.3390/cells14130989 (PMC12249355; doi:10.3390/cells14130989)
Supplement: Supplementary file 1 [file cells-14-00989-s001.zip › cells-3700338-supplementary.pdf]

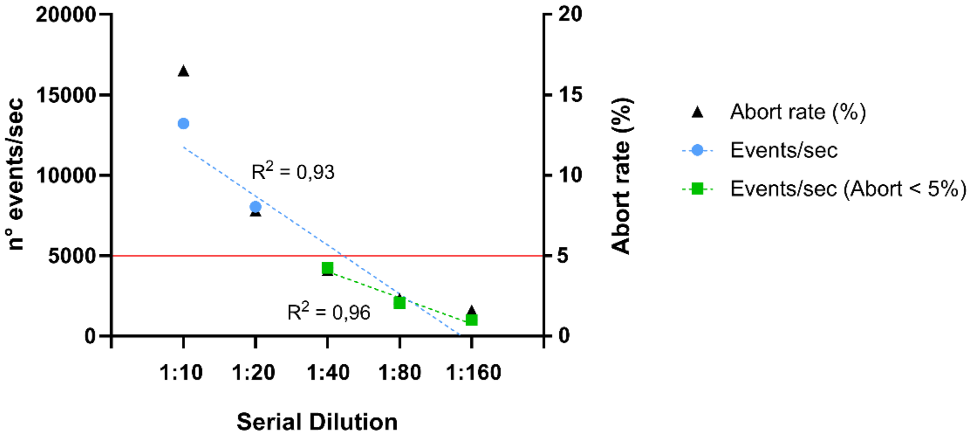

Figure S1. Accurate quantification of single Bodipy-exo detection. FC analysis of serial dilutions of Bodipy-exo showed a linear correlation between the sample concentration and the number of events/sec. When the sample concentration is too high and the abort rate exceeds 5% (red line), the swarm effect can occur, leading to an increase in clustered events and an underestimation of the actual number of Bodipy-exo in the sample.

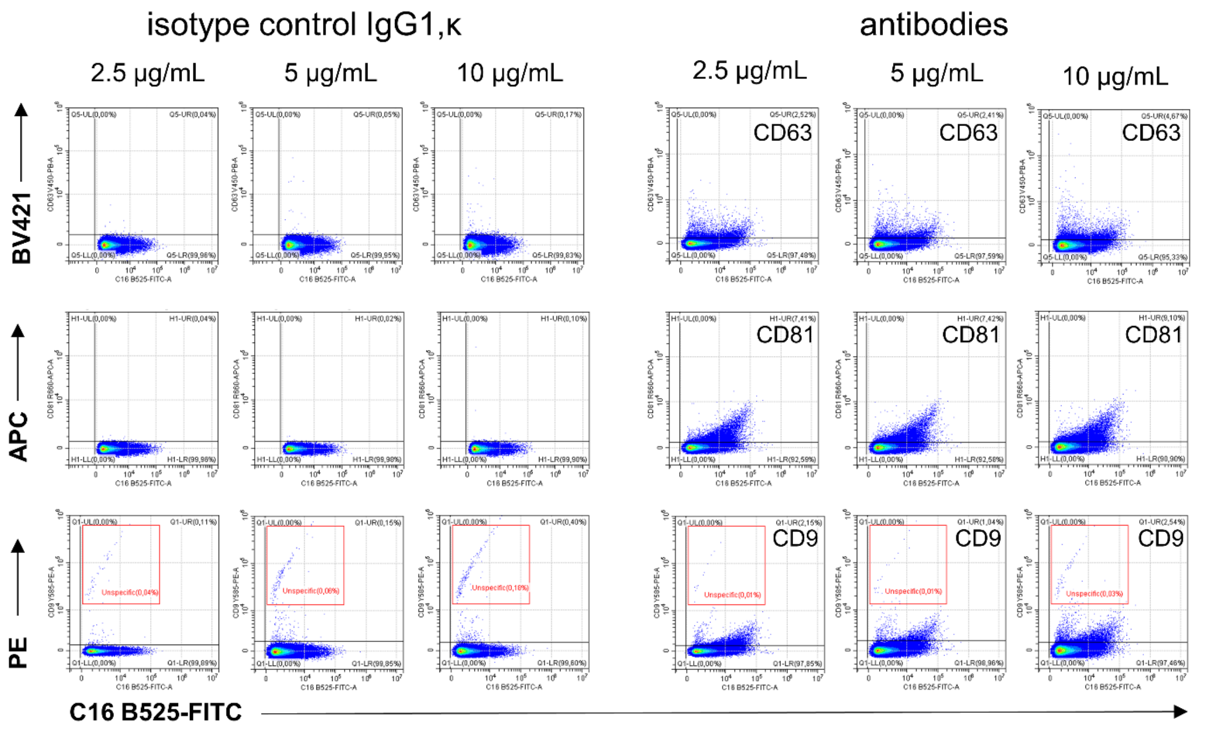

Figure S2. Isotype controls. A fixed number of Bodipy-exo (1×10^7) were stained with differed BV421 anti-CD63, APC anti-CD81 and PE anti-CD9 antibodies and their relative isotypes. The red rectangle indicates the background noise due to the PE-conjugated isotype that is subtracted to the percentage of colocalization between the antibodies and the Bodipy-exo.

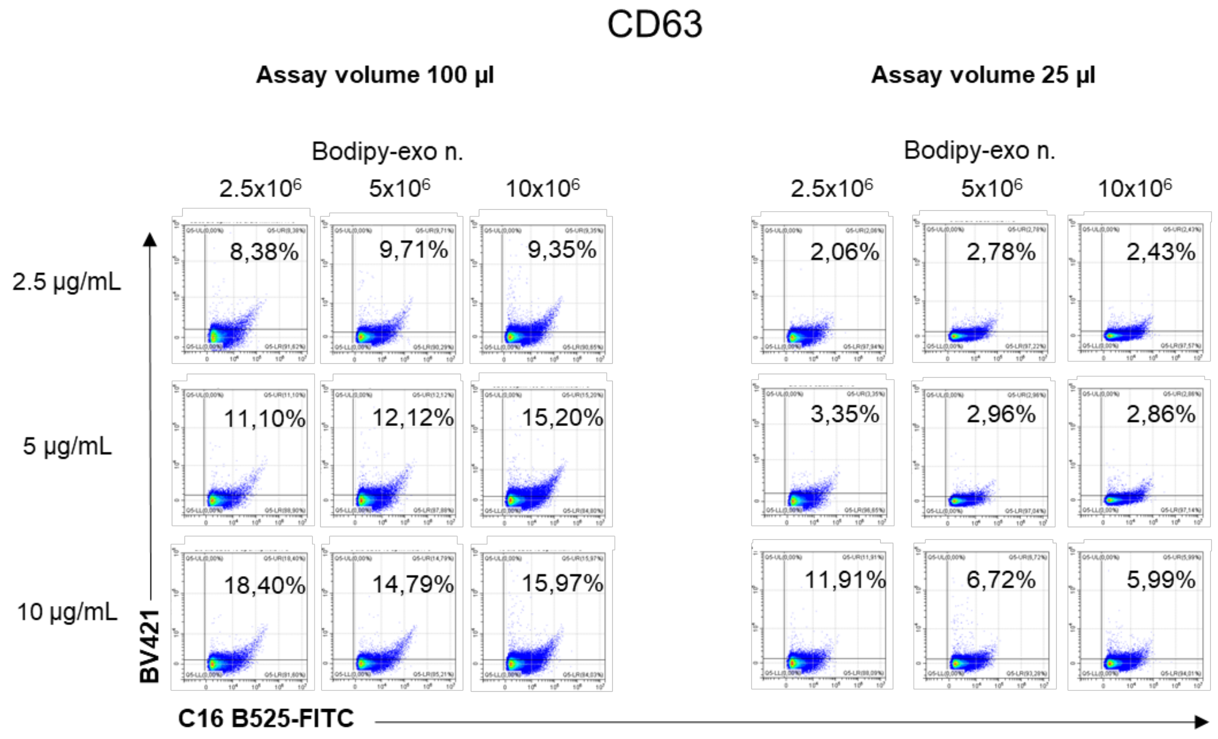

Figure S3. Comparison between assay volumes of 100 μL and 25 μL for CD63 staining. The assay performed in a low volume shows a decrease of the percentage of colocalization between the BV421-CD63 and the Bodipy-exo. In 100 μL a saturation of the labelling is reached at the concentration of 10 μg/ml of antibody.
